# TRPV2 channels facilitate pulmonary endothelial barrier recovery after ROS-induced permeability

**DOI:** 10.1016/j.redox.2025.103720

**Published:** 2025-06-07

**Authors:** Lena Schaller, Martina Kiefmann, Thomas Gudermann, Alexander Dietrich

**Affiliations:** Walther Straub Institute of Pharmacology and Toxicology, Member of the German Center for Lung Research (DZL), Medical Faculty, LMU Munich, Nußbaumstrasse 26, 80336, Munich, Germany

**Keywords:** TRPV2, ADAM10, ROS, Impedance, Cadherin, Endothelial barrier function

## Abstract

Reactive oxygen species (ROS), such as hydrogen peroxide (H_2_O_2_), are known signaling molecules that increase endothelial barrier permeability. In this study, we investigated the roles of redox-sensitive transient receptor potential (TRP) ion channels, TRPM2, TRPV2 and TRPV4, in H_2_O_2_-induced endothelial barrier dysfunction. Using primary human pulmonary microvascular endothelial cells (HPMEC), we employed impedance-based resistance measurements, Western blot, and immunofluorescence staining to assess the effects of H_2_O_2_ on the endothelial barrier. Exposure to sublytic concentrations of H_2_O_2_ caused an acute loss of endothelial barrier integrity, accompanied by the cleavage of vascular endothelial cadherin (VE-cadherin), which was also apparent after application of the TRPV2 activator cannabidiol. The inhibition of either TRPV2 with tranilast or a disintegrin and metalloprotease domain-containing protein 10 (ADAM10) with GI254023X significantly reduced H_2_O_2_-induced VE-cadherin cleavage, while TRPM2 inhibition by econazole significantly increased H_2_O_2_-driven VE-cadherin cleavage and blockage of TRPV4 showed no effect. Although inhibition of either TRPV2 or ADAM10 did not prevent the initial loss of barrier resistance upon H_2_O_2_ exposure, both were essential for the subsequent recovery of barrier integrity. Time-course immunofluorescence stainings revealed that HPMEC barrier recovery involved a transient localization of N-cadherin proteins at adherens junctions. This process of cadherin-switching did not occur upon inhibition of TRPV2 or ADAM10. Our results highlight a novel role for TRPV2 as a redox sensitive ion channels in the microvascular endothelium and provide insight into the mechanisms underlying pulmonary microvascular endothelial barrier recovery.

## Highlights

• H_2_O_2_-induced VE-cadherin cleavage is mediated by TRPV2/ADAM10 in lung endothelium.
• TRPM2 inhibition increased cleavage and TRPV4 inhibition had no effect.
• TRPV2 and ADAM10 are essential for recovery of barrier integrity.
• N-cadherin transiently localizes at adherens junctions during barrier recovery.

## Graphical abstract

**Figure:**
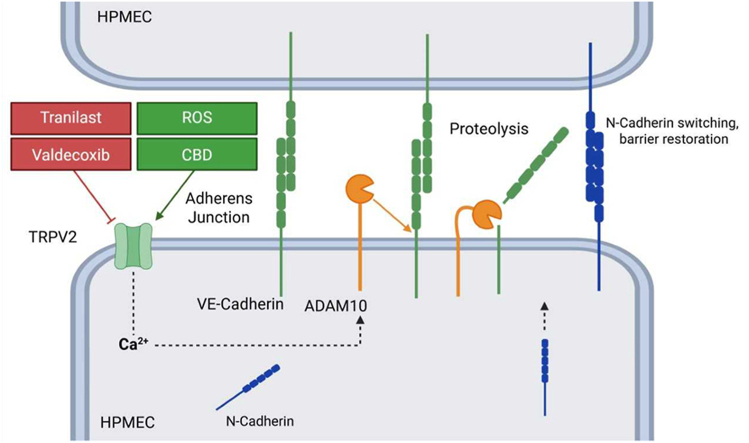

## 1 Introduction

The barrier formed by the pulmonary microvasculature is constitutively restrictive, preventing both pathogen infiltration and edema formation while facilitating the exchange of gases and nutrients between the bloodstream and surrounding tissue [1,2]. While a transient increase in permeability supports biological functions such as wound repair, angiogenesis and immune cell trafficking [3,4], prolonged or extensive permeability can result in pulmonary edema, acute respiratory distress syndrome (ARDS) [5] and atherosclerosis [6].

Reactive oxygen species (ROS), such as hydrogen peroxide (H_2_O_2_), are known effectors of altered endothelial barrier function [[7], [8], [9], [10]]. ROS can arise from exogenous triggers, including infection, ionizing radiation or toxicants, but also occur naturally in the body, such as during mitochondrial respiration [11,12]. The concept of an “oxidative window” describes the optimal range of ROS levels that facilitate cellular processes such as neovascularization, cell proliferation and wound healing [7,8,13]. Deviations from this balance, resulting in oxidative or reductive stress, lead to cellular dysfunction [8].

Adherens junctions (AJs), comprised of Ca^2+^-dependent, homotypic adhesions between the vascular endothelial cadherin (VE-cadherin) proteins of neighboring cells, are essential components of the endothelial barrier [3,14]. While the formation of AJs depends on extracellular Ca^2+^, an increase in intracellular Ca^2+^ can induce endothelial barrier permeability [3,15]. Members of the Transient Receptor Potential (TRP) superfamily form nonselective cation channels that conduct Ca^2+^, and several TRP channels have been implicated in Ca^2+^-induced barrier dysfunction [3]. It has been reported that TRP-induced Ca^2+^ influx could activate a disintegrin and metalloprotease domain-containing protein 10 (ADAM10) [16], a metalloprotease known to cleave VE-cadherin at its extracellular domain [17]. However, a ROS-driven, ADAM10-mediated cleavage of VE-cadherin has yet to be reported in pulmonary microvascular endothelial cells.

TRPM2 is a recognized mediator of ROS-induced Ca^2+^ influx. Expressed in the brain, immune cells, and vasculature, TRPM2 forms a tetrameric, nonselective ion channel that conducts Ca^2+^ and is gated by adenosine diphosphate ribose (ADPR) [[18], [19], [20], [21]], which is generated as a result of ROS-induced DNA damage [18,19]. While TRPM2 is a known modulator of pulmonary endothelial barrier permeability, its knockdown does not completely abolish endothelial Ca^2+^ influx following ROS exposure, suggesting the involvement of additional redox-sensitive Ca^2+^ channels [9,22].

The second member of the vanilloid TRP subfamily, TRPV2, is a potential candidate for the undefined source of ROS-induced pulmonary endothelial Ca^2+^ influx. Originally associated with mechanoreception [23], TRPV2 also operates as a redox-sensitive ion channel [24,25], and is highly expressed in the microvascular endothelium in relation to other redox-sensitive TRP channels, including TRPM2 and TRPV4 [26]. While TRPV2 has been linked to changes in blood-brain barrier integrity [27], there is no evidence to date linking the channel to altered pulmonary microvascular endothelial barrier function [28].

Here, we applied pharmacological inhibitors to investigate the role of TRPM2 and TRPV2 in H_2_O_2_-induced pulmonary endothelial barrier dysfunction. Neither channel was responsible for the initial loss of barrier resistance, but both channels facilitated the subsequent recovery of barrier integrity. In this model, TRPV2 mediated AJ integrity by inducing ADAM10-driven VE-cadherin cleavage, which was further increased upon TRPM2 inhibition. Endothelial barrier recovery was characterized by the translocation of neural cadherin (N-cadherin) to the plasma membrane, suggesting a role for TRP-mediated cadherin switching in the restoration of endothelial barrier function following ROS-induced permeability.

## 2 Methods

### 2.1 Cells

Primary human pulmonary microvascular endothelial cells (HPMECs) [29] from healthy donors were obtained from Promocell (Heidelberg, Germany, #C-12281) and cultured in endothelial cell growth medium MV (Promocell, #C-22020) at 37 °C and 5 % CO_2_, and were kept until passage 12. Donor information is provided in Supp. Table S1. Relevant ethical statements were provided by Promocell. For experiments involving pharmacological inhibition, HPMECs were pre-incubated for 1 h in DMEM containing the respective inhibitor(s), which were also present during the subsequent exposure period.

### 2.2 Quantification of endothelial barrier resistance

HPMECs were seeded onto electrical cell-substrate impedance sensing (ECIS) plates at a density of 8 × 10^4^ cells/well (Applied Biophysics, Troy, NY, USA, 8W10E+), which had been treated with 10 mM of l-Cysteine according to the manufacturer's recommendation. HPMEC barrier resistance was measured at 4000 Hz using the ECIS ZΦ device (Applied Biophysics), and experiments were conducted once the monolayer resistance had reached a constant state (after ∼48 h).

## 3 Results

### 3.1 H_2_O_2_ exposure at non-cytolytic concentrations increases HPMEC barrier permeability, triggers ADAM10-dependent VE-cadherin cleavage, and induces TRP-mediated Ca^2+^ flux

**Fig. 1:**
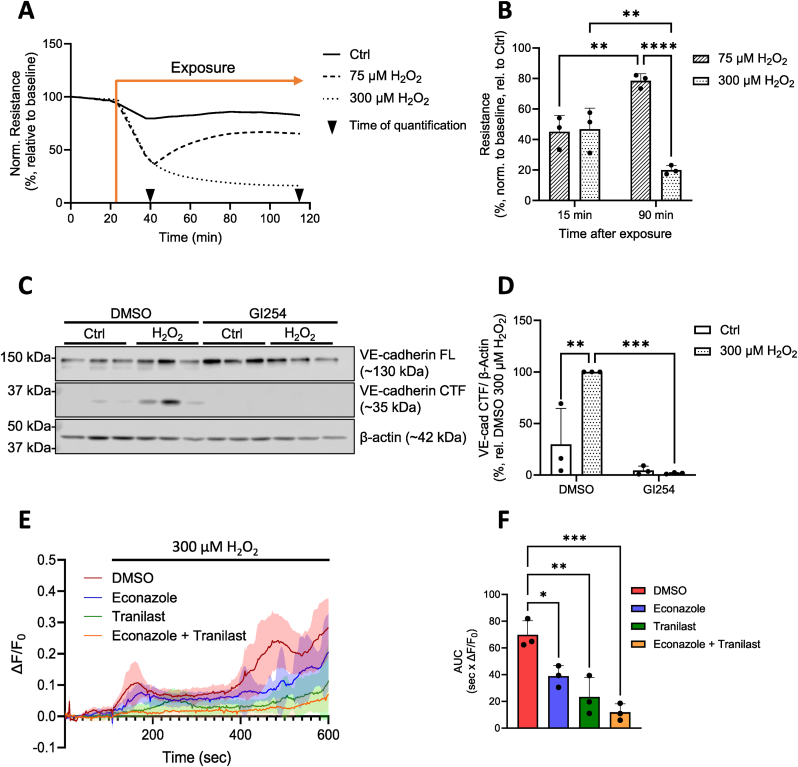
**H_2_O_2_ induces HPMEC barrier instability and ADAM10-dependent VE-cadherin cleavage.** Changes in HPMEC electrical resistance (normalized to baseline levels) were recorded with an ECIS device at 4000 Hz upon application of H_2_O_2_ (75 μM, 300 μM) (**A**). The normalized resistance values 15 and 90 min after exposure were quantified (**B**). Representative Western blot (from one donor, 3 technical replicates) of the full length (FL) and C-terminal fragment (CTF) levels of VE-cadherin protein in HPMECs 2 h after H_2_O_2_ exposure (300 μM) in the presence and absence of the ADAM10 inhibitor GI254023X (GI254, 3 μM) (**C**). β-actin was probed as a loading control. Normalized levels of VE-Cadherin CTF from these Western blots were quantified (**D**). Data reflect the mean (**A**, **B**, **D**) + SD (**B**, **D**) from 3 independent donors (*n* = 3). (**E**) Mean ΔF/F_0_ traces of HPMEC monolayer Ca^2+^ influx following H_2_O_2_ exposure (300 μM) in the presence and absence of the TRPM2 and TRPV2 inhibitors, econazole (10 μM) and tranilast (50 μM). Data represent the mean ± SD from one experiment, 35–50 cells/treatment group. This experiment was performed three times in HPMECs from a single donor at different passage numbers (*n* = 3), and the area under the curve (AUC) of each mean ΔF/F_0_ Ca^2+^ trace was quantified (**E**), with bars reflecting the mean + SEM. Normality of data was confirmed using the Shapiro-Wilk test, and significance between means was analyzed using two- or one-way ANOVA and Tukey post hoc tests (**B**, **D**, **E**); ∗*p* < 0.1, ∗∗*p* < 0.01, ∗∗∗*p* < 0.001, ∗∗∗∗*p* < 0.0001.

Using H_2_O_2_ to mimic ROS production in response to infection, radiation or other toxicants, we monitored changes in barrier resistance of human pulmonary microvascular endothelial cells (HPMEC). While H_2_O_2_ exposure did not exert detectable cytolytic effects after 2 h (Supp. Fig. S1), changes in HPMEC barrier resistance were observed within 5 min of exposure (Fig. 1A). 15 min post H_2_O_2_ addition, mean HPMEC barrier resistance dropped to 45 % ± 11 % and 47 % ± 14 % of the control in cells treated with 75 μM and 300 μM H_2_O_2_, respectively, with recovery noted only in HPMECs treated with 75 μM H_2_O_2_ (quantified in Fig. 1B). Additionally, Western blot analysis revealed that H_2_O_2_ exposure caused the formation of a single ∼35 kDa VE-cadherin C-terminal fragment (CTF) (Fig. 1C, Supp. Fig. S1B), the formation of which was prevented by the addition of the specific ADAM10 inhibitor GI254023X [30] (GI254, see Supp. Table S2 for IC_50_ values) (Fig. 1C, quantified in D). Cell fractionation through surface biotinylation revealed that this CTF was present as early as 15 min after exposure, and was detected at both the plasma membrane and the intracellular space (Supp. Fig. 1C). As ADAM10 is activated upon Ca^2+^ influx, we first turned our attention to two redox-sensitive TRP channels, TRPM2 and TRPV2. Quantitative rt-PCR confirmed that both genes were transcribed in HPMECs (Supp. Fig. S1D), and both proteins were detected in cell lysates via Western blot (Supp. Fig. S1E and F). Ca^2+^ imaging experiments revealed that the increase of intracellular Ca^2+^ ([Ca^2+^]_i_) upon H_2_O_2_ exposure was dependent on both channels (Fig. 1E and F).

### 3.2 TRPV2 mediates ADAM10-driven VE-cadherin shedding upon H_2_O_2_ exposure

**Fig. 2:**
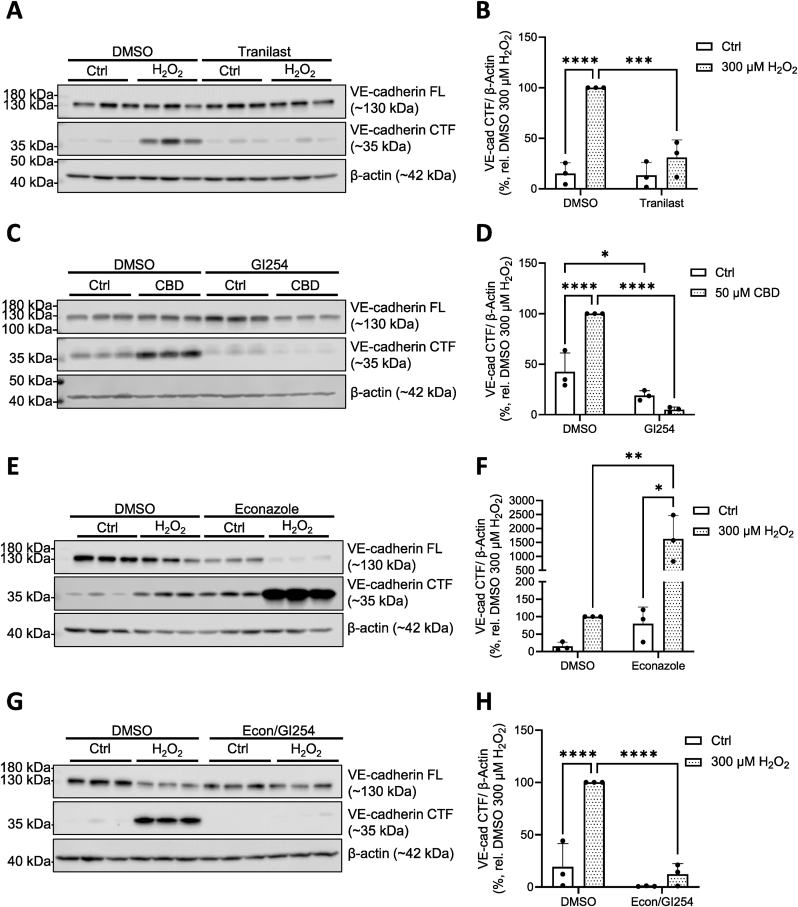
**TRPV2 and TRPM2 mediate VE-cadherin cleavage in HPMECs.** Representative Western blot of FL and CTF VE-cadherin protein levels in HPMECs upon TRPV2 inhibition (50 μM tranilast) and 2 h exposure to H_2_O_2_ (300 μM; **A**, quantified in **B**). (**C**) Representative Western blot of FL and CTF VE-cadherin protein levels in HPMECs upon ADAM10 inhibition (3 μM GI254023X, GI254) and 2 h exposure to cannabidiol (CBD, 50 μM), quantified in (**D**). Representative Western blot of FL and CTF VE-cadherin protein levels in HPMECs upon TRPM2 inhibition (10 μM econazole) and 2 h exposure to H_2_O_2_ (300 μM; **E**, quantified in **F**). Representative Western blot of FL and CTF VE-cadherin protein levels after H_2_O_2_ exposure (2 h, 300 μM) upon co-inhibition of TRPM2 and ADAM10 (10 μM econazole, 3 μM GI254023X, (**G**, quantified in **H**)). For all Western blots, β-actin was probed for as a loading control; samples shown are from a single donor, 3 technical replicates. Quantified data reflect the mean + SD from 3 independent donors (**B**, **D**, **F**) or 3 consecutive passages from one donor (**H**); (*n* = 3). Normality of data was confirmed using the Shapiro-Wilk test, and significance between means was analyzed using two-way ANOVA, with Tukey post hoc tests; ∗*p* < 0.05, ∗∗*p* < 0.01, ∗∗∗*p* < 0.001, ∗∗∗∗*p* < 0.0001.

Having demonstrated that H_2_O_2_ exposure triggers TRP-dependent Ca^2+^ influx, we next investigated the specific contributions of TRPV2 and TRPM2 to the associated VE-cadherin cleavage. HPMECs pretreated with the TRPV2 inhibitor tranilast [31] had significantly reduced H_2_O_2_-driven, ADAM10-mediated cleavage of VE-cadherin (Fig. 2A) when quantified (Fig. 2B). These results were corroborated using the alternate TRPV2 inhibitor valdecoxib [32] (Supp. Fig. S2A and B), as well as through siRNA-mediated TRPV2 knockdown (Supp. Fig. S2C and D). The TRPV2/ADAM10/VE-cadherin cleavage pathway was further confirmed with the TRPV2 activator, cannabidiol (CBD) [27] (Fig. 2C, quantified in D). Notably, while gene transcripts of the redox-sensitive TRPV4 channel were also detected in HPMECs (Supp. Fig. S1D), pretreatment with the specific TRPV4 inhibitor GSK2193874 [33] had no effect on the degree of H_2_O_2_–induced VE-cadherin CTF formation (Supp. Fig. S2E and F).

In contrast to TRPV2, we observed that the H_2_O_2_-induced VE-cadherin cleavage was more potent in the absence of TRPM2 functionality, as VE-cadherin CTF levels increased tenfold in HPMECs pretreated with the TRPM2 inhibitor econazole [34] (Fig. 2E, quantified in F). This finding was corroborated using the alternate TRPM2 inhibitor JNJ-28583113 [35] (Supp. Fig. S3A and B), as well as through siRNA-mediated TRPM2 knockdown (Supp. Fig. S3C and D). We observed that ADAM10 inhibition completely abolished H_2_O_2_-induced VE-cadherin CTF formation in econazole-treated HPMECs (Fig. 2G, quantified in H), suggesting that the absence of TRPM2 functionality exacerbates the TRPV2/ADAM10/VE-cadherin cleavage pathway. Further experiments into an underlying mechanism did not indicate direct involvement of TRPM2 in AJ destabilization, as H_2_O_2_-induced dephosphorylation of VE-cadherin was not altered in the presence of econazole (Supp. Fig. S3E and F). However, assays with the fluorigenic ROS probe H_2_DCFDA revealed that TRPM2 inhibition increased baseline intracellular ROS levels by 16.2 % ± 4.3 % relative to DMSO controls (Supp. Fig. S3G) within 30 min.

### 3.3 Recovery of HPMEC barrier integrity requires TRPV2 and TRPM2 functionality

**Fig. 3:**
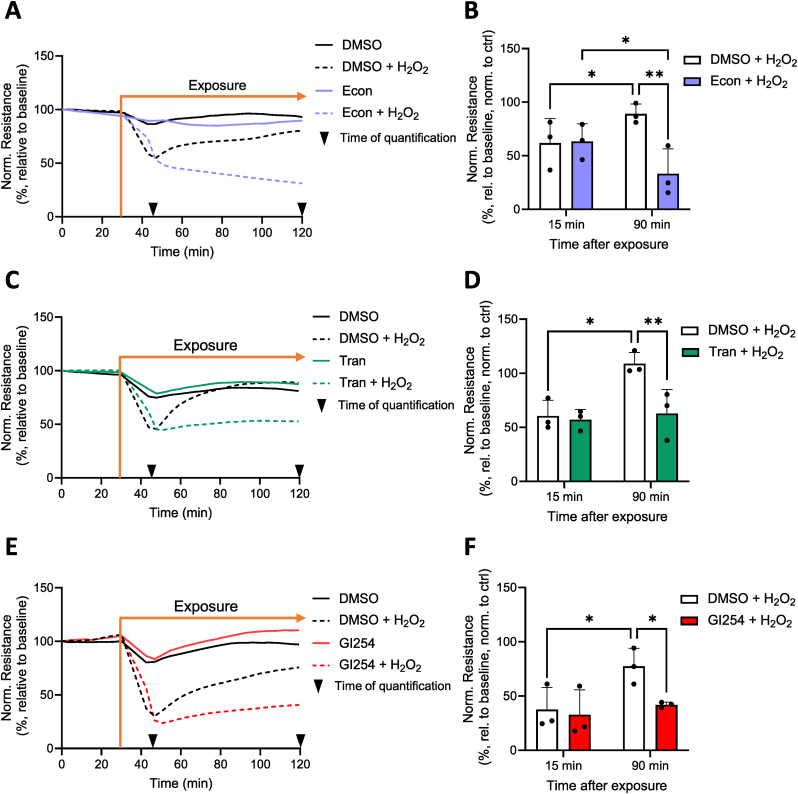
**TRPM2 and TRPV2 facilitate HPMEC barrier recovery following H_2_O_2_ exposure.** Changes in barrier resistance (normalized to baseline) were measured in HPMECs which were preincubated with DMSO or econazole (econ, 10 μM, 1 h) and subsequently exposed to 75 μM H_2_O_2_ (**A**). HPMEC resistance values (presented as % of control values) were quantified 15 and 90 min after H_2_O_2_ application (**B**). Similar experiments were conducted with the TRPV2 inhibitor tranilast (tran, 50 μM, 1 h preincubation, (**C**, **D**)) and the ADAM10 inhibitor GI254023X (GI254, 3 μM, 1 h preincubation, (**E**, **F**)). Data represent the mean (**A**–**F**) + SD (**B**, **D**, **F**) of results from 3 independent donors (*n* = 3). Normality of data was confirmed using the Shapiro-Wilk test, and significance between means was analyzed with two-way ANOVA and Tukey post hoc tests; ∗*p* < 0.05, ∗∗*p* < 0.01.

We next assessed whether inhibition of TRPV2 and TRPM2 channels would influence the HPMEC barrier response to H_2_O_2_. The protective effect of TRPM2 on VE-cadherin cleavage was also evident in our measurements of barrier resistance, as application of the TRPM2 inhibitor econazole prior to the addition of 75 μM H_2_O_2_ significantly impaired HPMEC barrier recovery (Fig. 3A), with resistance values dropping to 33 % ± 23 % of control values after 90 min of treatment (Fig. 3B). In addition to TRPM2, the TRPV2/ADAM10 axis was also necessary for HPMEC barrier recovery. HPMECs exposed to 75 μM H_2_O_2_ experienced significantly reduced recovery when pretreated with tranilast (Fig. 3C, quantified in Fig. 3D) or GI254023X (Fig. 3E, quantified in 3F).

### 3.4 TRPV2 facilitates HPMEC barrier recovery through “cadherin switching”

**Fig. 4:**
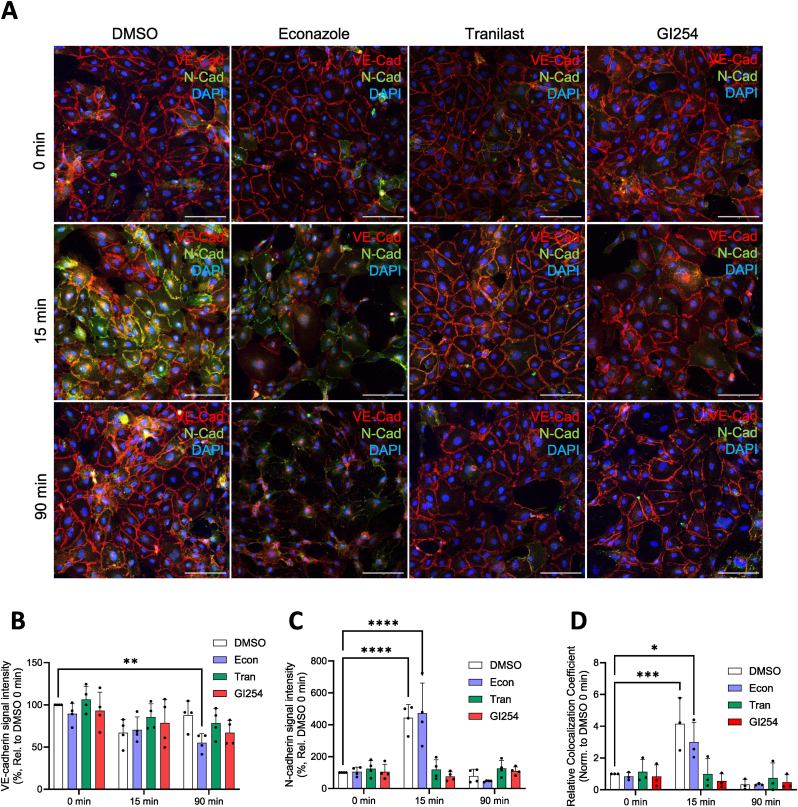
**TRPV2 and ADAM10 are necessary for altered localization of N- and VE-cadherin following H_2_O_2_ exposure.** (**A**) HPMEC immunofluorescence staining of VE-cadherin (red) and N-cadherin (green) over a timecourse of H_2_O_2_ exposure (75 μM; 0 min, 15 min, 90 min) in the presence and absence of TRPM2, TRPV2 or ADAM10 inhibitors (10 μM econazole, 50 μM tranilast, 3 μM GI254023X, respectively). Nuclei were stained with DAPI (blue), scale bars: 100 μm. Signal intensities of VE-cadherin (**B**) and N-cadherin (**C**) at cell-cell junctions were quantified from stainings performed in HPMECs from one donor at 4 consecutive passages (*n* = 4, 30 regions per *n*). Colocalization analyses for N- and VE-cadherin were conducted for the same regions in three experiments (*n* = 3, 30 regions per *n*), and mean weighted colocalization coefficients for VE-cadherin – N-cadherin were plotted (**D**). Normality of data was confirmed using the Shapiro-Wilk test, and significance between means (**B**–**D**) were analyzed with two-way ANOVA and Tukey post hoc tests; ∗*p* < 0.05, ∗∗*p* < 0.01, ∗∗∗*p* < 0.001, ∗∗∗∗*p* < 0.0001.

TRPV2-driven VE-cadherin cleavage could facilitate HPMEC barrier recovery by destabilizing AJs and enabling the translocation of neural cadherin (N-cadherin) to the plasma membrane, promoting wound healing. A time-course series of immunofluorescence stainings (Fig. 4A) revealed that, upon exposure to 75 μM H_2_O_2_, VE-cadherin signal became disorganized at the plasma membrane after 15 min, recovering within 90 min. In contrast, N-cadherin, while initially dispersed in the intracellular space, organized at the plasma membrane 15 min after H_2_O_2_ exposure, returning to the intracellular space within 90 min. N-cadherin may also be a target of ADAM10 ectodomain cleavage, as an H_2_O_2_-dependent ∼37 kDa N-cadherin CTF was detected in HPMEC lysates (Supp. Fig. 4). Quantification of VE-cadherin signal intensities at the borders of adjacent cells revealed that econazole-treated HPMECs showed a significant loss of VE-cadherin signal after 90 min of H_2_O_2_ exposure (Fig. 4B). 15 min after H_2_O_2_ exposure, N-cadherin signal intensities at the junctions of DMSO and econazole treated HPMECs were significantly elevated, while HPMECs pretreated with either TRPV2 or ADAM10 inhibitors showed no significant change in N-cadherin signal (Fig. 4C). Colocalization analyses of VE-cadherin and N-cadherin further confirmed that 75 μM H_2_O_2_ induced a transient localization of N-cadherin at AJs with VE-cadherin, a process that was significantly impaired upon either TRPV2 or ADAM10 inhibition (Fig. 4D).

## 4 Discussion

ROS are not only mediators of vascular pathology, but are also critical signaling molecules for endothelial cell proliferation, growth and motility [7,8,13,36]. In this study, we describe a pathway by which TRPV2 channels, alongside TRPM2 channels, modulate AJ protein composition and facilitate the recovery of HPMEC barrier function after H_2_O_2_ exposure.

We found that TRPV2, a redox-sensitive channel, played a significant role in mediating HPMEC response to ROS. H_2_O_2_ exposure triggered an [Ca^2+^]_i_ increase within 5 min, a reaction which was significantly reduced upon pharmacological inhibition of TRPV2. While the intracellular localization pattern of TRPV2 has not been determined in pulmonary endothelial cells, TRPV2 is rapidly translocated from internal stores to the plasma membrane upon application of insulin-like growth factor 1 (IGF-1) to CHO cells [37] or the chemotactic peptide fMetLeuPhe to macrophages [38]. Upon stimulation, TRPV2 localizes at the cell podosome [39], a membrane region of endothelial cells [40] that supports cell motility through localized proteolysis [41]. While we did not study the mechanisms of TRPV2 translocation, we observed that TRPV2 inhibition prevented the H_2_O_2_-driven proteolytic cleavage of VE-cadherin (Fig. 2A), a known substrate of the Ca^2+^-activated protease ADAM10 [17]. Therefore, we propose a novel TRPV2/ADAM10/VE-cadherin pathway through which HPMECs respond to ROS via ectodomain cleavage of VE-cadherin. Upon exposure to ROS, TRPV2 is activated, and the resulting Ca^2+^ influx activates the metalloprotease ADAM10, possibly through the Ca^2+^-activated scramblase, anoctamin 6 (ANO6) [42]. This cleavage event may not be restricted to ADAM10 and VE-cadherin, and the involvement of other Ca^2+^-activated proteases and their cell-adhesion substrates presents a promising avenue for further study. In addition, it remains to be determined whether the extracellular fragment of VE-cadherin released during this process plays a role in downstream signaling, as is the case with its epithelial counterpart E-cadherin [43].

Our study also offers insight into the complex function of TRPM2 in mediating vascular permeability. We observed that the initial H_2_O_2_-driven [Ca^2+^]_i_ increase in HPMECs was significantly reduced upon pharmacological inhibition of TRPM2, confirming the findings of previous studies [9,22]. In one such study, Mittal et al. described TRPM2-dependent changes in the VE-cadherin phosphorylation state at tyrosine residue 731, a site involved in VE-cadherin internalization [22,[44], [45], [46]]. Our results demonstrated that VE-cadherin Y731 was dephosphorylated within 5 min of H_2_O_2_ exposure, but that this process occurred independent of TRPM2 signaling. Our data suggest that, instead, TRPM2 facilitates HPMEC barrier recovery by maintaining cellular redox homeostasis, as has been described in interstitial macrophages [47], neutrophils [48], and myocytes [49]. As in these other cell types, we found that HPMECs pretreated with the TRPM2 inhibitor econazole had significantly elevated ROS levels compared to DMSO treated controls. This elevated oxidative stress in the absence of TRPM2 functionality could activate the TRPV2/ADAM10 pathway, which would account for the enhanced VE-cadherin cleavage observed under both baseline and H_2_O_2_-stimulated conditions upon TRPM2 inhibition. While both TRPM2 and TRPV2 mediate H_2_O_2_ –induced Ca^2+^ influx, our results demonstrate that the two channels serve different roles in HPMEC response to H_2_O_2._ This discrepancy could be attributed to channel localization and density, the study of which may be more feasible with the advent of novel specific antibodies and nanobodies. There is also the possibility that TRPV2 may facilitate localized Ca^2+^ influx, or “Ca^2+^ sparklets,” as has been described for TRPV4 in the vascular endothelium (reviewed in Ref. [50]).

The protective role of TRPM2 was also apparent in our measurements of HPMEC barrier resistance. Not only were HPMECs pretreated with econazole unable to recover their barrier integrity following 75 μM H_2_O_2_ exposure, but their barrier resistance continued to drop significantly over the course of 90 min. Unexpectedly, inhibition of the TRPV2/ADAM10 pathway also significantly limited the recovery of HPMEC barrier integrity after H_2_O_2_ exposure. One possible biological explanation for why a destructive process such as ectodomain cleavage could be beneficial for barrier recovery is to facilitate the process of “cadherin switching”. In the endothelium, VE-cadherin localizes primarily to AJs at the plasma membrane, where it is thought to contribute to contact inhibition of cell growth and proliferation [51,52]. N-cadherin, in contrast, is associated with cell migration and wound healing and is unable to translocate to the plasma membrane in the presence of VE-cadherin complexes at AJs [51,53]. Upon the disruption of VE-cadherin junctional organization, N-cadherin translocates to the cell surface, where it can form heterotypic adhesions with neighboring cells. The resulting N-cadherin adhesion complex promotes Rac1 activation, which in turn induces the reorganization of VE-cadherin at AJs [54]. We were able to observe this transient localization of N-cadherin at AJs in our timecourse immunofluorescence stainings, a process that did not occur upon inhibition of TRPV2 or ADAM10. Our results support a pathway by which redox-sensitive TRPV2 channels trigger the disruption of VE-cadherin dimers at HPMEC AJs through ADAM10-driven ectodomain cleavage. The resulting paracellular gaps are then rapidly repaired, possibly through N-cadherin-mediated recruitment of VE-cadherin. This pathway could be particularly relevant during leukocyte transmigration, which is characterized by H_2_O_2_ release [55], a temporary increase in paracellular permeability and a focal, transient loss of VE-cadherin complexes at AJs [4].

Future studies on the role of TRPV2 in pulmonary endothelial barrier function would benefit from the application of in vivo or ex vivo models. The isolated perfused and ventilated lung, for example, has been instrumental in toxicant screening [56], in highlighting TRPV4's role in ventilator-induced alveolar permeability [57], as well as in the investigation of H_2_O_2_-induced pulmonary edema [58,59]. Murine models could also give insight into the long-term transcriptional and translational regulation of the TRPV2/ADAM10 pathway. For example, mice exposed to cigarette smoke, an inducer of ROS and oxidative stress, showed reduced TRPV2 protein expression in alveolar macrophages [60]. It remains to be determined if this effect occurs in endothelial cells, but it is possible that TRPV2 and ADAM10-mediated VE-cadherin cleavage under chronic ROS exposure is kept in check through transcriptional regulation of TRPV2. Furthermore, additional histological studies on human biopsy tissue could provide more specific insight into the in situ membrane localization of TRPV2, which has previously been shown to differ from that of other TRP channels in epithelial cells [61]. Differing localization patterns could explain why TRPV2 was responsible for VE-cadherin cleavage in our HPMECs, in contrast to TRPV4, which has been reported to induce E-cadherin shedding in alveolar epithelial cells [62].

In summary, we confirmed that TRPM2 is only partially responsible for endothelial Ca^2+^ influx following oxidative signaling and identified TRPV2 as an additional redox-sensitive contributor. We described a novel signaling pathway by which TRPV2 activation alters AJ composition through ADAM10-mediated VE-cadherin cleavage and determined that this pathway is essential for endothelial barrier recovery following oxidative injury. These findings establish a foundation for future studies exploring redox-regulated pulmonary endothelial repair pathways in translationally relevant model.

## CRediT authorship contribution statement

**Lena Schaller:** Writing – review & editing, Writing – original draft, Methodology, Investigation, Formal analysis, Data curation, Conceptualization. **Martina Kiefmann:** Writing – review & editing, Supervision, Conceptualization. **Thomas Gudermann:** Writing – review & editing, Project administration. **Alexander Dietrich:** Writing – review & editing, Visualization, Supervision, Resources, Project administration, Methodology, Investigation, Funding acquisition, Formal analysis, Conceptualization.

## Data availability

All data are available in the main text or the supplementary materials.

## Funding

This study was supported by grants from the 10.13039/501100001659Deutsche Forschungsgemeinschaft (TRR152 (TG, 10.13039/100020014AD), and GRK 2338 (LS, TG, 10.13039/100020014AD), Deutsches Zentrum für Lungenforschung (10.13039/501100010564DZL) (TG, 10.13039/100020014AD), Germany.

## Declaration of competing interest

The authors declare that they have no known competing financial interests or personal relationships that could have appeared to influence the work reported in this paper.
