# Supplementary material for: TRPV2 channels facilitate pulmonary endothelial barrier recovery after ROS-induced permeability
Source: Redox Biol. 2025 Jun 7;85:103720. doi: 10.1016/j.redox.2025.103720 (PMC12210324; doi:10.1016/j.redox.2025.103720)
Supplement: Multimedia component 1 [file mmc1.pdf]

TRPV2 channels facilitate pulmonary endothelial barrier recovery after ROS-induced permeability

Lena Schaller, Martina Kiefmann, Thomas Gudermann, Alexander Dietrich*

Walther Straub Institute for Pharmacology and Toxicology, Member of the German Center for Lung Research (DZL), School of Medicine, LM University of Munich, Nußbaumstrasse 26, 80336 Munich, Germany.

*Corresponding author. Email: alexander.dietrich@lrz.uni-muenchen.de.

Supplementary Methods

Reagents and Antibodies

The following reagents and antibodies were used: High glucose Dulbecco's Modified Eagle Medium (DMEM, Thermo Fisher Scientific, Waltham, MA, USA, #41965039); hydrogen peroxide solution (Merck, Darmstadt, Germany, H1009); GI254023X (Tocris, Bristol, UK, #3995); econazole (Merck, Y0001236); JNJ-28583113 (MedChemExpress, Monmouth Junction, NJ, USA, #HY-149143); tranilast (Tocris, #1098); valdecoxib (Merck, #PZ0179); cannabidiol (Cayman Chemical, #90080, Ann Arbor, MI, USA); GSK2193874 (Tocris, #5106); anti-VE-Cadherin antibody (Cell Signaling, Danvers, MA, US, #2500); anti-phospho-VE-Cadherin (Tyr731) antibody (Thermo Fisher Scientific, #441145G); anti-N-Cadherin antibody (BD Biosciences, Franklin Lakes, NJ, US, #610920); horseradish peroxidase (HRP)-conjugated anti-β-actin antibody (Merck, #A3854); anti-TRPM2 antibody (Bethyl, Montgomery, TX, USA, #A300-414A); anti-TRPV2 antibody (Abcam, Cambridge, UK, Ab272862); peroxidase (POX)-conjugated anti-rabbit antibody (Merck, #A1654); horseradish peroxidase (HRP)-conjugated anti-mouse antibody (Cell Signaling, #7076); goat anti-rabbit IgG Alexa Fluor 488 (Thermo Fisher Scientific, #A-11008); goat anti-mouse IgG Alexa Fluor 594 (Thermo Fisher Scientific, #A11005). See complete antibody information in Supp. Table S3.

SiRNA knockdown of TRPV2 and TRPM2

Pools of TRPV2 siRNAs (100 nM, ON-TARGETplus, Horizon Discovery, Cambridge, UK, #L-004194-00-0050), TRPM2 siRNAs (30 nM, ON-TARGETplus, Horizon Discovery, #L-004193-00-0005), or nonspecific control siRNAs (ON-TARGETplus, Horizon Discovery, #D-001810-10-50) were introduced to HPMECs through reverse transcription using the DharmaFECT 2 transfection reagent (Horizon Discovery, #T-2002-03), per manufacturer's instructions. Media was replaced after 24 h, and cells were allowed to grow to confluency over a period of 3 days, at which point cells were lysed for RNA isolation or treated and harvested for Western blotting. Knockdown efficacy was determined through qRT-PCR and Western blotting.

Cell Surface Biotinylation and Cell Fractionation

HPMEC surface proteins following H_2O_2 exposure were isolated through surface biotinylation. Briefly, following exposure, HPMECs were washed once with ice cold PBS and incubated with the cell membrane impermeable biotinylation reagent EZ Link™ Sulfo-NHS-SS-Biotin (1 mg/ml, Thermo Fisher Scientific, #21217) for 1 h on ice. HPMECs were then washed with cold PBS, incubated with 50 mM glycine in PBS for 15 min to quench excess biotin, and subsequently washed twice in cold PBS. Cells were then lysed in 150 µl RIPA buffer (with protease and phosphatase inhibitors) for 1 h on ice. Lysates were spun at 14,000 rpm, 4 °C for 30 min. 50 µl of supernatant was added to 17 µl of streptavidin-conjugated Dynabeads™ (Thermo Fisher Scientific, #11206D), and rotated overnight at 4 °C. The following day, biotin/bead bound proteins were magnetically separated from the unbiotinylated fraction, washed thrice in RIPA, and resuspended in RIPA with 1x Laemmli buffer (prepared from 5x stock: 3 ml TRIS/HCl (2.6 M), pH 6.8; 10 ml glycerin; 2 g SDS; 2 mg bromophenol blue; 5 ml β-mercaptoethanol). Samples for both fractions were then assessed using Western blot.

SDS-PAGE and Western blot

The expression of VE-cadherin protein was evaluated by Western blot analysis as previously described [S1]. Following treatment, HPMECs were lysed in 150 µl RIPA buffer (with protease and phosphatase inhibitors) for 30 min on ice. Protein concentration was quantified with the Pierce BCA Protein Assay Kit (Thermo Fisher Scientific, #23225) according to the manufacturer's protocol. Protein samples (10-30 µg lysate, 1x Laemmli buffer (prepared from 5x stock: 3 ml TRIS/HCl (2.6 M), pH 6.8; 10 ml glycerin; 2 g

SDS; 2 mg bromophenol blue; 5 ml β-mercaptoethanol)) were heated for 10 min at 95 °C and loaded onto an SDS-PAGE gel (4 % stacking, 10 % separating). SDS-PAGE gel electrophoresis was run for 30 min at 80 V, and then at 120 V for 90 min. Proteins were then transferred from the gel to a Roti®-PVDF membrane (Roth, Karlsruhe, Germany, #T830.1) in a wet transfer system (BioRad, Feldkirchen, Germany) at 50 V for 1.5 h. The membrane was then blocked with 5 % low-fat milk (Roth, #T145.2) in TBS-T (0.1 % Tween20) for 1 h at RT. All antibodies were diluted in the milk blocking solution. See Supp. Table S3 for relevant antibody information. Membranes were incubated in the primary antibody solutions overnight at 4 °C. Afterwards, membranes were washed (3 x 10 min, TBS-T) and incubated for 2 h at RT in peroxidase-conjugated secondary antibody solutions. Chemiluminescence was detected following incubation in SuperSignal West Femto or Pico maximum sensitivity substrates (Life Technologies, CA, USA, #34095 and #34580), using an Odyssey-Fc unit (Licor, Lincoln, NE, USA). Uncut Western blot images for all samples and replicates can be found on the online Open Science Foundation repository, OSF DOI: 10.17605/OSF.IO/T3FJP.

Ca^{2+} Imaging

HPMECs were grown on poly-L-lysine-coated 24 mm glass coverslips until 80 % confluency. On the day of measurement, HPMECs were loaded with 2 µM Fura-2-AM (Merck, #47989-1MG-F) in Ca^{2+} buffer (0.1 % BSA in HBSS (with Ca^{2+}, Mg^{2+} and 0.5 M HEPES)) for 25 min at 37 °C. Coverslips were then washed with HEPES/HBSS buffer, placed into a quick-change chamber (Warner instruments, Holliston, USA, #64-0367) with 450 uL HEPES/HBSS, and positioned on the 40x oil-objective of a Leica DM98 fluorescence microscope. Changes in intracellular Ca^{2+} concentration following the application of H_2O_2 (300 µM, Merck, #H1009) were recorded at 340 and 380 nm wavelengths, as described [S1]. For measurements involving pharmacological inhibition, the respective inhibitors were included in both the Fura incubation solution and the treatment solutions.

Immunocytochemistry

HPMECs were seeded on poly-L-lysine-coated 12 mm glass coverslips. After treatment, cells were washed once with cold PBS, fixed in 4 % PFA/PBS (15 min, RT), and then washed thrice with cold PBS. Cells were permeabilized for 10 min at RT in a 0.2 % Triton X-100/PBS solution, and then washed 4 x 5 min in PBS-T (0.1 % Tween20 in PBS). HPMECs were blocked for 1 h in PBS with 0.1 % Tween20 and 5 % BSA, and then incubated overnight at 4 °C in primary antibody solutions prepared in blocking buffer. See complete antibody information in Supp. Table S3. The following day, cells were washed (4 x 5 min, PBS-T), incubated for 2 h at RT in secondary antibody solutions, and washed again (4 x 5 min, PBS-T). All antibodies were diluted in blocking buffer. Nuclei were stained with DAPI (0.1 mg/L in PBS) for 3 min at RT, after which cells were washed (4 x 5 min, PBS-T). Coverslips were mounted with PermaFluor mounting medium (Epredia, Kalamazoo, MI, USA, #TA-030-FM) and kept at 4 °C. Confocal images were taken with a Zeiss LSM 880 microscope (Zeiss, Oberkochen, Germany) using the ZEN Black software (Zeiss, version 2.3). Images were processed with FIJI software (Image J v.1.53c, Wayne Rasband, NIH, USA) [S2]. Signal quantification was performed with ZEN Blue and Black software (Zeiss, version 3.4). Regions of interest (ROI) were drawn along the borders of adjacent HPMECs. Mean N-cadherin and VE-cadherin signal intensity values from ten ROI were calculated for 3 images from each condition and timepoint. Colocalization analyses were conducted following the guidelines recommended by the software provider. Briefly, single-label control samples were used to set the gating for the experimental, double-labeled samples. VE-cadherin/N-cadherin colocalization coefficients were determined for ten ROI per image, with 3 images taken for each condition and timepoint. These immunocytochemistry experiments were replicated thrice in subsequent passages from one donor.

LDH Cytotoxicity Assay

H_2O_2-induced cytotoxicity was assessed through an LDH assay (Merck, #11644793001), according to the manufacturer's protocol. Briefly, cells were incubated in 300 µM H_2O_2 for 2h, at which point the supernatant was collected and tested for the reduction of tetrazolium salt by NADH as a measure of LDH activity. Triton X100 (2 %) was applied as a positive control.

Quantitative Reverse-Transcription (qRT)-PCR

Total RNA from HPMECs was isolated with the RNeasy Plus Mini Kit (Qiagen, Hilden, Germany, #74136). 1 µg mRNA was then transcribed to cDNA using the RevertAid H Minus First Strand cDNA Synthesis Kit (Life Technologies, Darmstadt, Germany, #K1631), with reverse transcription polymerase and random primers according to the manufacturer's protocol. The levels of mRNA transcripts of target genes were assessed using real-time quantitative PCR, as described previously [S1]. Briefly, 3 µl (15 ng) of template cDNA was added to 7 µl of a master mix containing 2x Absolute QPCR SYBR Green Mix (Life Technologies, #AB1158B), 10 pmol of the respective primer pair (Metabion, Planegg, Germany, see table S4 for primer sequences) and water. For qRT-PCR, the following program was run in a light-cycler 480 device (Roche, Mannheim, Germany): activation (15 min, 94℃); 45 cycles of

denaturation (12 s, 94℃), annealing (30 s, 50℃) and extension (30 s, 72℃); and melting curve analysis. The default lightcycler software (Roche, Basel, Switzerland) allowed for the calculation of crossing points (Cps), which were used to calculate gene expression values.

Quantification of Reactive Oxygen Species (ROS)
The cell-permeable, fluorigenic probe 2', 7'-dicholodihydrofluorescein diacetate (H_2DCFDA) was used to semi-quantitatively assess ROS levels in HPMECs, as previously described [44]. HPMECs were plated at a density of 15,000 cells/well in 96 well plates and grown to confluency over 24-48 hrs, at which point HPMECs were pretreated with H_2DCFDA (50 µM, Thermo Fisher Scientific, #3135794) for 1 h in DMEM without phenol red. Cells were then washed once in warm DMEM without phenol red (Thermo Fisher Scientific, #21063029), and incubated with DMSO or econazole (10 µM) in DMEM without phenol red for indicated time points. Fluorescence emitted by the oxidized DCF was detected using a microplate reader (Fluostar Omega, BMG labtech, Ortenberg, Germany). L-Cysteine (2 mM, Merck, #168159) was applied as an antioxidant negative control, and phorbol-12-myristat-13-acetate (PMA, 20 µM) served as a positive control of intracellular ROS production.

Statistical analysis
Statistical analysis was performed with GraphPad Prism 10 software (GraphPad Software, San Diego, USA). Significant differences are indicated by asterisks, where $p < 0.05$ (*), 0.01 (**), 0.001 (***), and 0.0001 (****).

Supplementary References

S1. Hofmann, K.; Fiedler S.; Vierkotten S.; Weber J.; Klee S.; Jia J.; Zwickenpflug, W.; Flockerzi, V.; Storch, U.; Yildirim AÖ.; Gudermann T.; Königshoff M, Dietrich A. Classical transient receptor potential 6 (TRPC6) channels support myofibroblast differentiation and development of experimental pulmonary fibrosis. *Biochim Biophys Acta Mol Basis Dis.* **2017**, *1863*, 560-568, doi: 10.1016/j.bbadis.2016.12.002.

S2. Schindelin, J.; Arganda-Carreras, I..; Frise, E.; Kaynig, V.; Longair, M.; Pietzsch, T.; Preibisch S.; Rueden, C.; Saalfeld, S.; Schmid, B.; Tinevez, JY.; White, DJ.; Hartenstein, V.; Eliceiri, K.; Tomancak, P.; Cardona, A. Fiji: an open-source platform for biological-image analysis. *Nat Methods* **2012**, *9*, 676–682, doi: 10.1038/nmeth.2019.

Supplementary Tables and Figures

Table S1: HPMEC donor information (Promocell)

Donor ID (Lot #)	Catalogue #	Age	Sex	Ethnicity	Disease Status
467Z025.1	C-12281	61	Female	Caucasian	Healthy
463Z013.1	C-12281	57	Female	Caucasian	Healthy
489Z006.1	C-12281	51	Male	Caucasian	Healthy
489Z005	C-12281	52	Female	Caucasian	Healthy

Table S2: IC_{50} values for TRP and ADAM inhibitors

Compound	Target	Expression System	Assay Type	IC_{50}
Econazole	TRPM2	HEK293, hTRPM2	Electrophysiology	< 3 µM [34]
JNJ-28583113	TRPM2	HEK293, hTRPM2	Electrophysiology	126 nM [35]
Tranilast	TRPV2	HEK293T, hTRPV2	Fluorometric Assay	2.3 µM [31]
Valdecoxib	TRPV2	HEK293, rtTRPV2	Fluorometric Assay	9 µM [32]
GI254023X	ADAM10	COS-7, hADAM10	Enzymatic Cleavage Assay	5.3 nM [30]
GSK2193874	TRPV4	HEK293T, hTRPV4	Fluorometric Assay	2 nM [33]

Table S3: Antibodies used for Western blotting and immunocytochemistry

Primary Antibodies	Supplier	Cat. # / RRID	Dilution
VE-Cadherin (rb pAb)	Cell Signaling	2158 / AB_2077970	WB: 1:1,000; ICC: 1:400
Phospho-VE-cadherin (Tyr731) (rb pAb)	ThermoFisher Scientific	44-1145G / AB_2533584	WB: 1:1,000
N-Cadherin (Mo pAb)	BD biosciences	610920 / AB_2077527	WB: 1:1,000; ICC: 1:400
TRPM2 (rb pAb)	Bethyl	A300-414A / AB_2208495	WB: 1:300
TRPV2 (rb pAb)	Abcam	Ab272862 / AB_2892218	WB: 1:300
B-actin-HRP (mo pAb)	Merck	A3854 / AB_262011	WB: 1:10,000

Secondary Antibodies	Supplier	Cat. # / RRID	Dilution
Rabbit IgG-POX	Merck	A6154 / AB_258284	WB: 1:10,000
Mouse IgG-HRP	Cell Signaling	7076 / AB_330924	WB: 1:10,000
Rabbit Alexa Fluor 488	ThermoFisher Scientific	A32731 / AB_2633280	ICC: 1:250
Mouse Alexa Fluor 594	ThermoFisher Scientific	A-11005 / AB_2534073	ICC: 1:250

Table S4: DNA-sequences of qRT-PCR primer pairs

Gene	Forward Primer	Reverse Primer
TRPC1	GAG AGC ATT TGA ACT TAG TGC TGA	TTA CAT TGC CGG GCT AGT TC
TRPC4	GGT CAG ACT TGA ACA GGC AAG	GTT TAA TTT CTC CCC ATA TGA AGC
TRPV2	CTG ACC GTT GGC ACT AAG C	CTC CCA TGA AGC CCA GTT C
TRPV4	GGA CAC GTG TGG GGA AGA	CAC AGC CAG CAT CTC GTG
TRPM2	GCC TCA GCT GCT TCG G	CTT CAC CAC CAG CAC TTC CA
TRPM7	AGA CTC GGC TTC TGC TGC TA	TCC AGG ATT TCT GGG ACA TTC TC

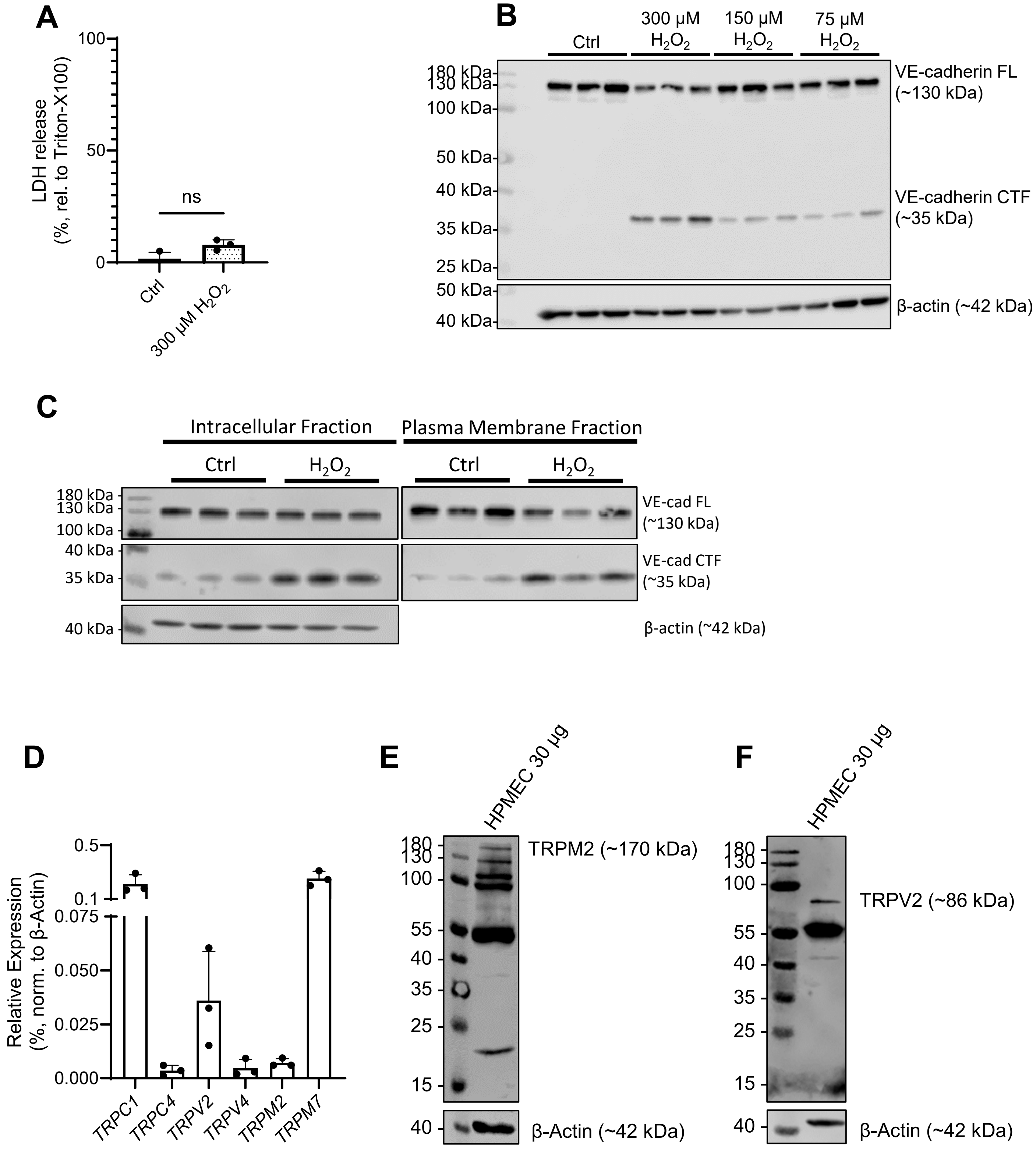

Fig. S1. Characterization of HPMECs and HPMEC response to H_2O_2. (**A**) Degree of cytolysis, measured in terms of lactate dehydrogenase (LDH) activity, in HPMECs after 2 h H_2O_2 exposure (300 µM). Data represent the mean + SD of results from 3 independent donors (*n* = 3). Significance was assessed using a Wilcoxon test. ns = no significance. (**B**) Complete Western blot of the full length (FL) and C-terminal fragment (CTF) levels of VE-cadherin protein in HPMECs 2 h after H_2O_2 exposure at varying concentrations (75 µM, 150 µM and 300 µM). β-actin was probed as a loading control. Data represent 3 technical replicates from one donor (*n* = 1). (**C**) Representative Western blot of VE-cadherin FL and CTF protein levels after 15 min H_2O_2 exposure (300 µM). The intracellular and plasma membrane fractions of HPMEC protein lysates were separated through extracellular biotin labeling and streptavidin selection. β-actin was probed as a loading control. Data represent 3 technical replicates from one donor (*n* = 1). (**D**) TRP gene expression results, as detected by qRT-PCR, normalized to β-actin. Data reflect mean values + SD from 3 independent donors (*n* = 3). TRPM2 (**E**) and TRPV2 (**F**) proteins were detected in HPMEC lysates by Western blot, and β-actin was probed as a loading control.

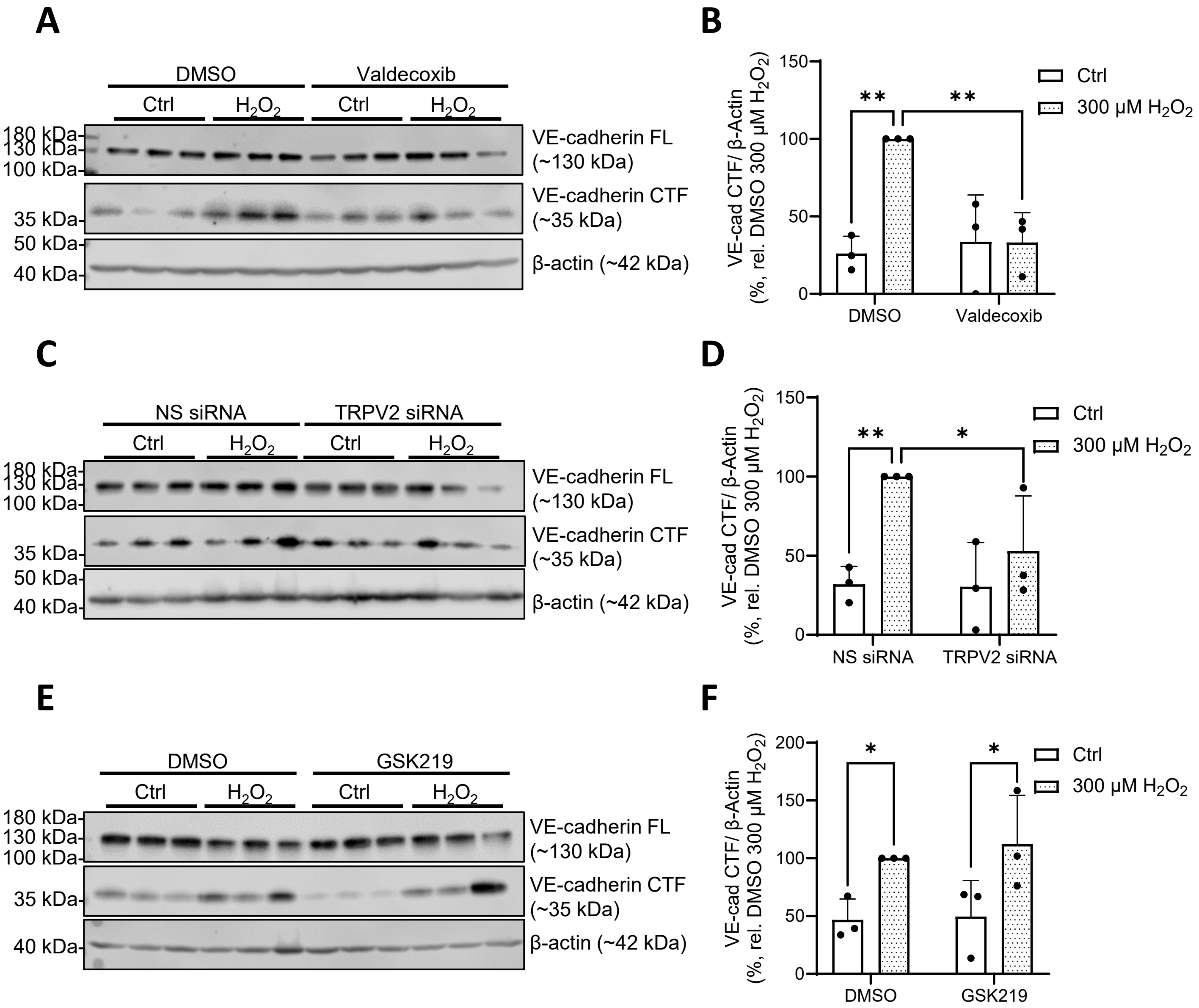

Fig. S2. Additional controls for TRPV2 modulation of HPMEC VE-cadherin upon H_2O_2 exposure. Representative Western blots of FL and CTF VE-cadherin protein levels after H_2O_2 exposure (2 h, 300 µM) upon TRPV2 inhibition (100 µM valdecoxib, (**A**, quantified in **B**)) or siRNA-mediated TRPV2 knockdown (100nM siRNA, NS = Nonspecific control, (**C**, quantified in **D**)). (**E**) Representative Western blot of FL and CTF VE-cadherin protein levels after H_2O_2 exposure (2 h, 300 µM) upon TRPV4 inhibition (300 nM GSK2193874, quantified in **F**). For all Western blots, β-actin was probed for as a loading control, samples shown are from a single donor, 3 technical replicates. Western blot quantifications represent the mean + SD of results from 3 independent donors (**B**) or 3 consecutive passages from one donor (**D**, **F**); ($n = 3$). Normality of data was confirmed using the Shapiro-Wilk test, and significance between means was analyzed using two-way ANOVA, with Tukey post hoc test; * $p < 0.05$, ** $p < 0.01$.

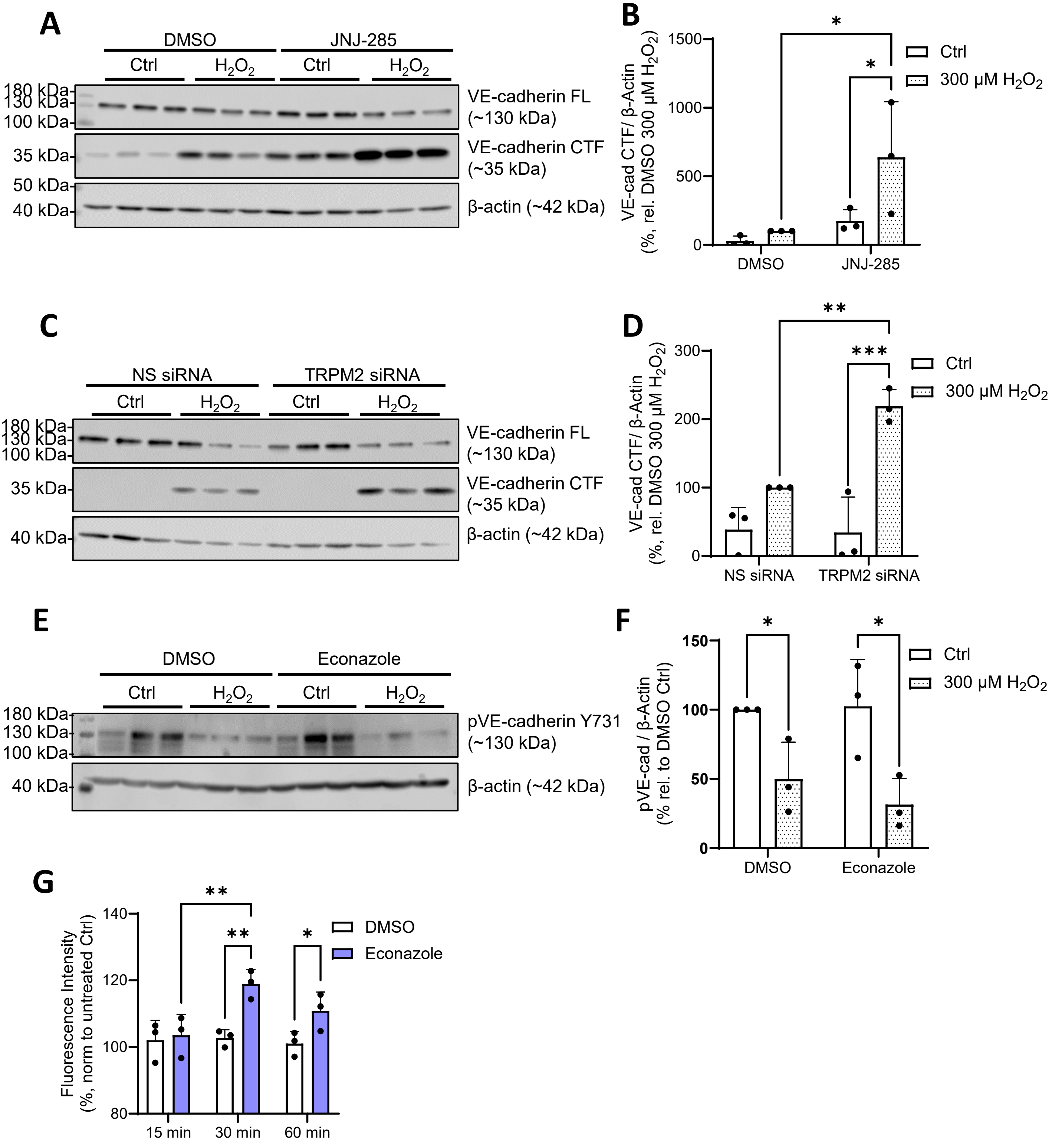

Fig. S3. Impaired TRPM2 functionality increases ADAM10-mediated HPMEC VE-cadherin cleavage. Representative Western blots of FL and CTF VE-cadherin protein levels after H_2O_2 exposure (2 h, 300 µM) upon TRPM2 inhibition (10 µM JNJ-28583113, (**A**, quantified in **B**)) or siRNA-mediated TRPM2 knockdown (30 nM siRNA, NS = Nonspecific control, (**C**, quantified in **D**)). Representative Western blot of phosphorylated VE-cadherin (pY731) protein levels after H_2O_2 exposure (5 min, 300 µM) upon TRPM2 inhibition (10 µM econazole, (**E**, quantified in **F**)). For all Western blots, β-actin was probed for as a loading control, samples shown are from a single donor, 3 technical replicates. Western blot quantifications represent the mean + SD of results from 3 independent donors (**B, D**) or 3 consecutive passages from one donor (**F**); ($n = 3$). (**G**) Detection of HPMEC ROS levels through the fluorigenic ROS probe H_2DCFDA after incubation with DMSO or econazole (10 µM) for the described timepoints. Data reflect the mean + SD of results from one donor at 3 consecutive passages ($n = 3$). Normality of data was confirmed using the Shapiro-Wilk test, and significance between means was analyzed using two-way ANOVA, with Tukey post hoc test; * $p < 0.05$, ** $p < 0.01$, *** $p < 0.001$.

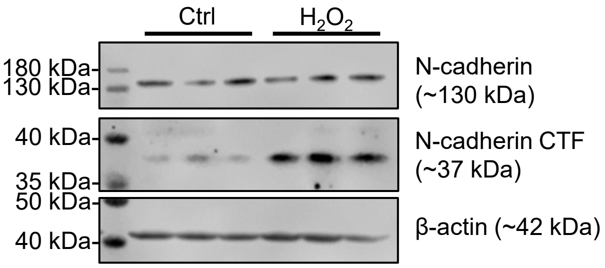

Figure S4. H_2O_2 exposure induces N-cadherin cleavage in HPMECs. Representative Western blot of FL and CTF N-cadherin protein levels after H_2O_2 exposure (2 h, 300 μM). β-actin was probed for as a loading control, samples shown are from a single donor, 3 technical replicates; (*n* = 1).
